# Cyanobacterial Polyhydroxyalkanoates: A Sustainable Alternative in Circular Economy

**DOI:** 10.3390/molecules25184331

**Published:** 2020-09-22

**Authors:** Diana Gomes Gradíssimo, Luciana Pereira Xavier, Agenor Valadares Santos

**Affiliations:** 1 Post Graduation Program in Biotechnology, Institute of Biological Sciences, Universidade Federal do Pará, Augusto Corrêa Street, Guamá, Belém, PA 66075-110, Brazil; 2 Laboratory of Biotechnology of Enzymes and Biotransformations, Institute of Biological Sciences, Universidade Federal do Pará, Augusto Corrêa Street, Guamá, Belém, PA 66075-110, Brazil; lpxavier@ufpa.br

**Keywords:** biopolymer, biorefinery, cyanobacteria, circular economy, polyhydroxyalkanoate, waste

## Abstract

Conventional petrochemical plastics have become a serious environmental problem. Its unbridled use, especially in non-durable goods, has generated an accumulation of waste that is difficult to measure, threatening aquatic and terrestrial ecosystems. The replacement of these plastics with cleaner alternatives, such as polyhydroxyalkanoates (PHA), can only be achieved by cost reductions in the production of microbial bioplastics, in order to compete with the very low costs of fossil fuel plastics. The biggest costs are carbon sources and nutrients, which can be appeased with the use of photosynthetic organisms, such as cyanobacteria, that have a minimum requirement for nutrients, and also using agro-industrial waste, such as the livestock industry, which in turn benefits from the by-products of PHA biotechnological production, for example pigments and nutrients. Circular economy can help solve the current problems in the search for a sustainable production of bioplastic: reducing production costs, reusing waste, mitigating CO_2_, promoting bioremediation and making better use of cyanobacteria metabolites in different industries.

## 1. Introduction

An urgent demand for biotechnology is to find alternatives to conventional plastics, derived from hydrocarbons, which are harmful to the environment not only in its exploration and refining, but also in its disposal. In 2018, more than 359 million tons of plastic was produced worldwide [1]. Since traditional plastic is not biodegradable, it depends on human action for its degradation, however a very small portion of fossil plastic is actually recycled. About 35.4 million tons of plastic is discarded annually by the United States alone, and only an estimated 8.4% is sent for recycling [2]. In the last 50 years, we have primarily and almost exclusively depended on petrochemical plastics, due to its wide range of applications and cheap manufacture; for example, in 1995, a kilo of polypropylene cost less than US $1.00 to produce, which justifies the predilection for this type of polymer [3].

The consequences of its unbridled use are already visible and have been studied for a long time. Since the 1970s, researchers have been warning about the high prevalence of microplastics, the result of the wear of fossil fuel plastics with a size of less than 1 mm, in marine environments and its damage to this ecosystem [4]. Alternatives to conventional plastics are being studied and some are already on the market, these polymers can be classified as polynucleotides, polyamides, polysaccharides, polyoxoesters, polythioesters, polyphosphates, polyisoprenoides and polyphenols [5]. We focus here on polyhydroxyalkanoates (PHA), especially polyhydroxybutyrates (PHB), examples of polyesters, which have similar applications as polypropylene with physical-chemical characteristics comparable to this petrochemical plastic [6]. In addition to its favorable structural properties, this thermoplastic of natural origin is biodegradable, water resistant and liable to be manipulated by techniques that are already widespread in the industry, such as injection, being better absorbed by current industrial equipment [7].

An alternative has to be found, one that does not produce non-biodegradable waste such as petrochemical residues with its high molecular masses accumulating in the soil and water for a long period of time [8,9]. Despite the environmental advantages of PHA over conventional plastics, for its replacement to be a reality, it is necessary to reduce the costs associated with the microbiological production of these biopolymers. The main obstacle in the process is the carbon source used to maintain fermentation costs, the yield of the chosen entries, the productivity and the downstream processing, including purification [10,11].

**Figure 1:**
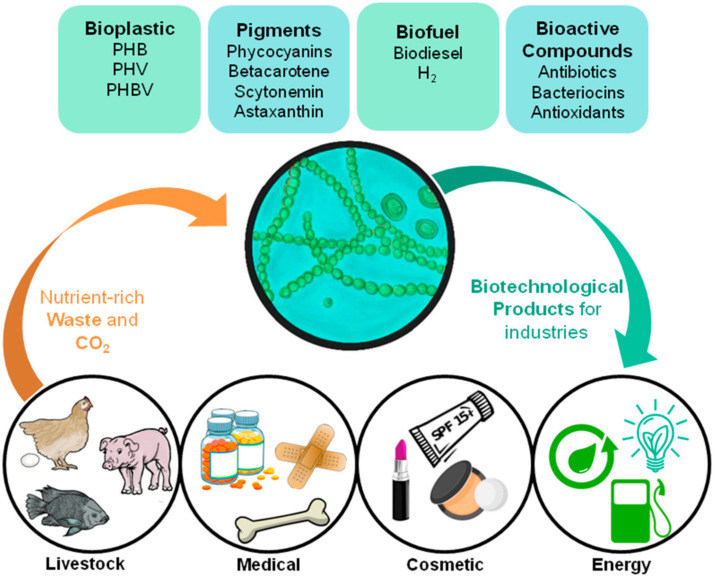
Diagrammatic representation showing cyanobacteria’s role in a circular economy-based system for various industries, and its possible products and waste assimilation.

The use of cyanobacteria as industrial PHA producers makes it possible to reduce the cost of nutritional inputs, since these photosynthetic organisms have fewer nutritional needs than heterotrophic bacteria [12,13]. The potential application of cyanobacteria by-products in industries with high added value [14,15] is interesting from an economic and environmental point of view, even more so if this system is implemented in light of the circular economy (Figure 1).

In this review, we seek to demonstrate the feasibility of applying the concept of circular economy in the production of PHA by cyanobacteria, a strategy that has been proposed for microalgae in general, including eukaryotic algae, and notably, for the production of biofuel [16,17,18,19,20]. We present an introduction to bioplastics, focusing on PHA, its biosynthesis, properties and applications. With respect to cyanobacteria, we bring PHB production by some species, and in the hopes of taking advantage of their potential to lower industrial PHB production costs, we also show the effectiveness of these organisms as bioremediators and waste nutrient removers. With the current work, we aim to add to the knowledge of this field, which is still somewhat deficient, in order to make the substitution, or at least reduction of petrochemical plastics, a more attainable goal.

## 2. Bioplastics

There is still controversy over the term “bioplastic” as there is still no standardized definition [21]. In this review, we embrace the two most broadly used definitions for this environment-friendly plastic: (1) bioplastics are polymers originated either entirely or in part from renewable natural sources, according to the Organization for Economic Co-operation and Development (OECD) [22], bioplastic can be viewed as synonymous with bio-based, and in addition to generating cleaner residues in its production, their decomposition is less harmful than that of petrochemical plastics, and its wear time is also considerably shorter [23,24,25]. The second definition (2) takes into account its biodegradability, as in German norm EN13432 [26], which refers not to the origin of the polymer, but to its ability to be degraded by organisms such as fungi, bacteria and algae [27,28]. The PHA and PHB addressed here are both bio-based and biodegradable.

Some of the most promising types of bioplastic are polysaccharides, such as starch and cellulose, and polyesters, including PHA [29]. When speaking of bioplastics used in packaging, worldwide, the two polysaccharides mentioned correspond to 30.7% of the global market for bio-based packaging—22.2% starch and 8.5% the representative portion of cellulose. As for polyesters—totaling 50.6%—the largest portion is of poly lactic acid plastic (PLA), 42.5%, followed by 6.7% of aliphatic and aromatic co-polyesters (AA) and 1.4% of PHA [30]. In terms of values, the global plastic packaging market was already estimated at 6.1 billion dollars in 2015, and the sector is expected to increase its value to more than 25 billion by 2022. The packaging segment is still the main use for plastics of natural origin, corresponding to the destination of 58% of all bioplastics produced in 2017. Next, we have the textile industry with 11% and the automobile and consumer goods industries with 7% each [31].

An important sector of application of bioplastics, especially PHA, is in medicine and the pharmaceutical industry, in the manufacture of prostheses, surgical material, as a scaffold in tissue engineering and used in drug carriers [23,32]. A characteristic that makes these biotechnological applications of PHA possible is the biocompatibility of these polymers, which can be implanted in the body without causing inflammation [33,34].

### 2.1. Polyhydroxyalkanoates

PHA are neutral lipids stored in the cells of cyanobacteria and other organisms as an energy reserve and carbon source. They are thermostable and elastomer bioplastics that have physical properties similar to plastics of fossil origin [23,35]. They are produced from the microbial fermentation of sugars, lipids, alkanes, alkenes and alkanoic acids and stored and accumulated in granules in the cytoplasm [36], with its occurrence extending to some archaea and bacteria, both Gram-negative and Gram-positive, and with no apparent prevalence in any specific phylum. This accumulation of polymers is not limited to taxonomic realms, nor to environmental niches, occurring in both terrestrial and aquatic organisms [37,38].

The main natural sources for obtaining PHA, especially different types of PHB, are heterotrophic bacteria, achieving good yields, with accumulation of PHB up to 85% dry cell weight (dcw) in *Cupriavidus necator* [39]. In addition to the good yields obtained, these species proved to be competent in assimilating alternative carbon sources for the production of PHA, using vinasse and molasses from sugar production and even waste frying oil [40,41]. Due to the relative ease in using genetic engineering with these bacteria, several studies deal with the production by recombinant organisms or even heterologous expression in *Escherichia coli*, with good results, such as the yield of 70–90% (dcw) [42,43,44]. Marine prokaryotes have a high production of PHA, with accumulation of up to 80% of its dry weight in bioplastic, and this good performance is observed in organisms that inhabit areas rich in nutrients [45], corroborating with what was proposed in the 1950s, regarding the role of excess carbon sources in bacterial biopolymer production [46].

The predilection for research and development of bioplastic production by heterotrophic bacteria is justified by the considerable production and accumulation of PHA in their cells, with some species such as *Cupriavidus metallidurans* and *E. coli*, wild or genetically modified, being used for the production of PHA on a larger scale [47,48]. Despite the good production of biopolymer by these strains, an operational problem hinders its use at an industrial level: the need for more elaborated carbon sources, which can compete with the food sector [49], and are expensive with the cost of nutritional inputs reaching up to 50% of production costs [50]. 

One way to mitigate these high costs in cultivation is using renewable and cheap carbon sources, such as domestic and industrial waste, which applies to heterotrophic bacteria [40,41,48] as well as photosynthetic organisms, that require fewer nutrients for their growth and production of biomass and biotechnological metabolites, such as bioplastic [51,52], thus, producing biopolymers basically from light and CO_2_ [14]; in this context, we can understand the potential of the so-called blue algae, the cyanobacteria.

Perhaps the main advantage of these polymers, so versatile in their characteristics and applications, is the ability to be degraded naturally. Being of biological origin, these plastics are biodegradable, and they can be digested by PHA-depolymerases. The degradation of PHA biofilms at 25 °C in soil, sludge or seawater is in the range of 5 to 7 µm per week [23], thus being an attractive alternative to petrochemical plastics and an important research focus. The decomposition of PHA, either by the action of bacteria or natural elements, depends on some factors, such as the composition of the polymer, and the temperature and humidity of the environment, which also helps accelerate this process. In the case of microbial degradation, the decomposing microorganism also influences the degradation time, since different bacteria, for example, express different PHA-depolymerases, enzymes responsible for the degradation of the biopolymer [53]. The physical and chemical characteristics of PHA cause them to sink in aquatic environments, which also favor its conversion to carbon and water by decomposers [47].

PHA are gaining visibility as possibilities for replacing petrochemical plastics in an increasingly tangible way. Many stages of its production have not been improved yet, but much has been achieved in this area of research since the 1920s, when studies began on this biopolymer with the detection and extraction of poly(3-hydroxybutyrate) (P(3HB) or PHB) from *Bacillus* sp. [6,54,55]. The structural variations of a biopolymer, such as different monomers, will result in different physical-chemical characteristics and mechanical properties, which make different PHA more or less suitable for a given application.

#### 2.1.1. PHA Structure

**Figure 2:**
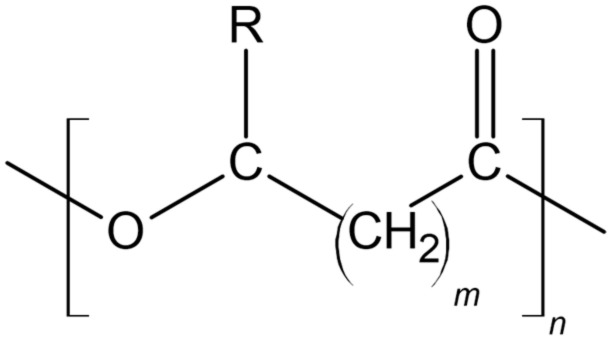
Polyhydroxyalkanoates (PHA) general structure, where *m* ranges from 1 to 3, with 1 being most common, as in polyhydroxybutyrates (PHB), *n* is the degree of polymerization with values from 100 to 30,000, and the variable *R* is the alkyl group with different chain lengths and structures in PHB. *R* = methyl.

Polyhydroxyalkanoates are linear polyesters with a basic structure (Figure 2) formed by 3 to 6 hydroxy acids [56]. PHA polymers and copolymers can contain more than 150 monomers, reaching a molecular weight of up to 2 million Daltons [34]. The production of these polymers occurs in microorganisms through the use of substrates such as alcohols, sugars and alkanes, and the different chemical structures give polymers different physical properties, which may be more suitable for certain applications [23,56].

These polymers can be separated into three categories, according to the number of carbons, short chain length (SCL), with 3 to 5, medium chain length (MCL), formed by 6 to 14 carbons, and PHA with more than 14 carbons are called long chain length (LCL) [57]. Different organisms and bacteria genera produce different polyesters [47], and a determining factor in the production of PHA is the carbon source used by the producing organism, which can result in vastly different chemical structures. Some microbial products are said to be related to their carbon source, i.e., they are similar to the input used, and others are called unrelated, as they differ from the raw material consumed, presenting, for example, a different number of carbons [56]. PHA can also be presented as homo-polymers, such as PHB, or copolymers, like the poly(3-hydroxybutyrate-co-3-hydroxyvalerate) (PHVB), depending on the structural variation of its monomers [37].

#### 2.1.2. PHA Biosynthetic Pathways

How microorganisms assimilate different forms of carbon into different polymers occurs through three biosynthetic pathways. The first, and most well-known pathway for PHA production, especially PHB, the one most found in cyanobacteria, is well described in archaea and heterotrophic bacteria as in the freshwater bacillus *Cupriavidus metallidurans*, and other species of the same genus. These granules are the result of a metabolic process that has two acetyl-CoA molecules as a precursor, derived from the tricarboxylic acid cycle (TCA) [13,58,59]. The reversible condensation of these two molecules is mediated by β-ketothiolase (encoded by gene *phaA*), the intermediate generated is reduced by the action of an NADPH-linked acetyl-CoA reductase (*phaB*), resulting in d(-)-3-hydroxybutyryl-CoA, which is then polymerized by the action of PHA polymerase (*phaC* and *phaE*), generating the poly(3-hydroxybutyrate) biopolymer (PHB) [17].

The second biosynthetic pathway uses lipid metabolism, and its medium-chain PHA product is based on the biotransformation of alkanes, alkenes and alkanoates, and the carbon source is directly related to the product’s monomeric composition [60,61]. This production occurs through the β-oxidation pathway of fatty acids, in which different hydroxyalkanoate monomers are generated and then polymerized using PHA synthase enzymes [59,60,61,62]. An important step in this path is the conversion of trans-2-enoyl-CoA, a β-oxidation intermediate, to (R)-hydroxyacyl-CoA, an R-specific enoyl-CoA hydratase, encoded by *phaJ*. This enzyme acts in an (R)-specific manner and has already been reported in *Aeromonas caviae* and *Pseudomonas putida* [60,63].

The last biosynthesis pathway produces alkanoate monomers that also result in medium chain length PHA (MCL-PHA); however, the precursors of this pathway are simple carbon sources such as sucrose, glucose and fructose, which makes it a potentially less expensive industrial process [6,23]. In this pathway, we have the presence of both sugars and lipids, starting from a glycolic precursor and making use of fatty acid biosynthesis intermediates. The Entner–Doudoroff pathway is used by *Pseudomonas* sp., with the catalysis of the sugar source from glucose to pyruvic acid [64]. This feat is made possible by the action of PHA synthase which catalyzes the biosynthesis of PHA from fatty acids as well as from sugars [65]. As in the second pathway, a key enzyme in this reaction is an (R)-specific, acyl-ACP-CoA transacylase, encoded by the *phaG* gene [66].

In addition to the carbon source, other nutrients such as nitrogen, phosphate, sulfur, oxygen, or deprivation of these, can affect growth and also play an important role in PHA biosynthesis [67,68]. Changes in the C:N ratio have been used in culture optimization, including evaluation of waste as a nutritional source [46,69,70], with abundance of carbon being favorable to biomass production [71] and limiting phosphorus and/or nitrogen as most beneficial stress for PHA production [72,73]. Such strategies have shown good results: in the extremophilic archea *Haloferax mediterranei*, an accumulation of 47.22% (dcw) of PHB was found using a 1:35 ratio of C:N. This higher value found for PHB production differs from the best C:N condition for the production of extracellular polymeric substances (EPSs) in *H. mediterranei*, which was 1:5, this result is interesting because it allows to direct production to the biopolymer of interest according to the nutritional medium [74]. An increase in the ratio between the two nutrients resulted in an even greater accumulation, 1:125 of C:N, providing an accumulation of up to 59% (dcw) of PHB using activated sludge from food industry waste [68].

**Figure 3:**
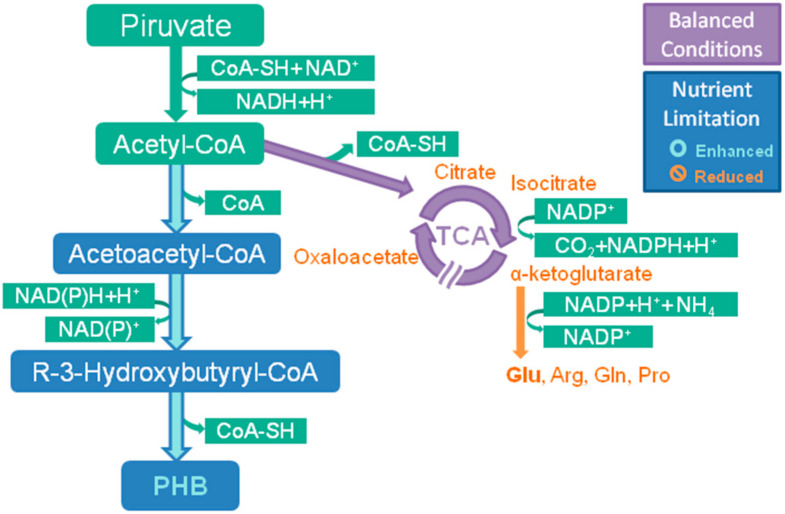
Carbon flow under balanced culture conditions, in purple, showing flux towards tricarboxylic acid cycle and under nutritional stress due to nitrogen and/or phosphorus limitation, enhanced flux or accumulation in blue and reduced activity in orange, with the carbon flux being directed to PHB biosynthesis.

Nitrogen is a micronutrient present in proteins and nucleic acids and its limitation can affect these metabolisms [36,75], also influencing the concentration of NAD(P)H and the ratio NAD(P)H/NAD(P) within the cell [58,59]. Whereas in a balanced culture (Figure 3), without nutritional stress, the concentration of these co-factors remains constant since the flow in TCA is maintained, in a situation of nitrogen deprivation, the synthesis of amino acids is reduced, and the decrease in the conversion of a-ketoglutarate into glutamate, which assimilates ammonium ions into the cell causing an accumulation of NAD(P)H [76,77], can then be used to reduce acetoacetyl-CoA to R-3-hydroxybutyryl-CoA. Another interesting supplement for the accumulation of NADPH is citrate, since it reduces citrate synthase activity in the Krebs cycle, supplementation in *A. fertilissima* increased PHB production compared to control [78], and citrate synthase can also be inhibited by high concentrations of NAD and NADPH [58].

Phosphorus, in its inorganic form as adenosine triphosphate, acts in protein and nucleic acid syntheses in addition to being important in the assimilation of lipids and carbohydrates and other roles in cell maintenance [36,39]. Despite the need for a minimum concentration of phosphorus in the cell to guarantee vital functions, the limitation of this nutrient proved to be a good strategy for inducing the production of PHA, and it is sometimes more significant as a limiting factor than nitrogen in cyanobacteria [77]. Nutritional stress due to phosphorus limitation also results in changes in TCA, restricting the Krebs cycle [73]. In balanced nutritional conditions, there is a greater amount of coenzyme-A (CoA-SH), which inhibits the synthesis of PHA. The withdrawal of nutrients like phosphorus and nitrogen from the metabolism promotes the accumulation of NADH, since citrate synthase and isocitrate dehydrogenase are inhibited with a consequent increase in acetyl-coA [36,59,79], the initial molecule for PHA biosynthesis [13,76].

#### 2.1.3. Physical and Mechanical Properties

The chemical structure of PHA, such as number of monomers, directly influences the physical characteristics of the polymer as well as its mechanical properties. While shorter PHA have high crystallinity and are quite brittle, polymers with a greater number of monomers can be more elastic and flexible [39,80]. The composition of the biopolymer also determines its degradation: in medical applications, for example, this determines whether its rate of degradation is compatible with a patient’s tissue regeneration [81,82]. 

In order for biopolymers to replace conventional plastics, they must play the same role. For this, they must have similar physical and mechanical properties, in order to be inserted in different industries without major losses and ideally being assimilated by the same machinery already installed, producing it through techniques such as injection molding and extrusion [83]. PHB has a range of Tm (melting temperature), which refers to the average melting temperature, similar to 190 °C of polypropylene and 176 °C of LDPE (Low-Density Polyethylene), that is, it supports similar temperatures. It is also comparable to polypropylene in its limit of tensile strength, which is the force necessary to stretch a material until it breaks [37,83]. However, the low value of Tg (glass transition temperature) of PHB makes for poor flexibility. 

Another parameter in which PHB does not match petrochemicals is in fracture elongation, the mechanical property a material has to be stretched, in terms of percentage over the original size of the material. While the two conventional plastics mentioned above reach up to 620% of its size before rupture, PHB supports only up to 6%. This shows the importance of tailor-made PHA in order to adjust the properties of a polymer to the desired parameter, the most suitable for its purpose [84,85]. P(4HB), for example, which has two monomeric units instead of just one like PHB, manages to reach up to 1000% of its initial size before breaking [6], with the added advantage of a greater UV rays’ resistance [86].

Mechanical properties can vary widely even among PHA, short chain length PHA (SCL-PHA), for example, are quite brittle thanks to their high crystallinity, and the addition of 3-hydroxyvalerate units enhances copolymer flexibility [87,88]. To further illustrate the relation between structure and proprieties, the addition of 20 mol% of PHV in the polymer P(3HB-co-20 mol% 3HV) make it more malleable but also more heat-sensible than pure PHB, enduring temperatures up to 150 °C, and no higher than 180 °C. Increasing the PHV content to 71 mol% in P(3HB-co-71 mol% 3HV) reduces its Tm even more, to only 83 °C [85]. Regarding the physical and mechanical properties of poly(3-hydroxybutyrate-co-3-hydroxyhexanoate) (P(3HB-co-3HHx)), which is already produced on an industrial scale in 20,000 L reactors by heterotrophic bacteria, namely *Aeromonas hydrophila* [89], its rapid degradation by PHA depolymerases can be attributed to its lower crystallinity [90]; hence, this copolymer can be well-used in disposable products. Its thermo-tolerance and overall mechanical characteristics, such as 150% enhancement in stiffness, were improved with the addition of alfalfa and hemp fibers forming a composite [91]. This approach is another way of benefiting natural polymers. For example, composite production using natural fibers with the incorporation of 30% *w*/*w* of pineapple leaf fibers with PHBV increased the polymer tensile strength by 100% [6,85]. Other mixtures show good results, like the greater impact resistance and heat tolerance achieved by blending PHB with polycaprolactone (PCL) [92].

The tailor-made modeling of PHA choosing each of its monomers or in blends with other polymers, including synthetic ones, is a way to improve the physical properties of a bioplastic and it can also reduce production costs [93,94]. This can be achieved with simple changes in cultivation, using block polymers as an example in the heterotrophic bacteria model *C. necator,* P(3HB) was obtained using fructose as a substrate, and it was possible to add 3-hydroxyvalerate units by applying supplementation with pentanoic acid pulses in a bioreactor, thus changing its physical properties [95,96]. Recombinant organisms can also be used for the production of block copolymer, with the copolymer consisting of poly-3-hydroxybutyrate (PHB) as one block, and random copolymer of 3-hydroxyvalerate (3HV) and 3-hydroxyheptanoate (3HHp) as another block, obtained with a *Pseudomonas putida* KTOY06ΔC (*phaPCJAc*), where the PHA synthesis gene *phaPCJAc* was cloned from *Aeromonas caviae* [97], resulting in improved mechanical properties.

#### 2.1.4. Applications

The structural diversity of PHA makes it an attractive alternative in numerous industrial sectors, and different applications can benefit from different aspects of PHA, whether it be its biocompatibility, biodegradability or its natural origin, which make it cleaner compared to hydrocarbon plastics. The most obvious, and most explored, use of PHA is in the production of packaging and non-durable goods, taking advantage of its biodegradability. The first consumer good using PHA was launched to the public in the 1990s, with a line of shampoos and conditioners with a PHA packaging, made with BIOPOL, a copolymer of PHB and PHV, with good water resistance [45]. Since then, other companies have shown interest in the study and production of PHA, for example, the German company Biomer, which through research with *A. latus*, managed to produce and accumulate up to 90% (dcw) in PHB using commercial sucrose as feedstock [37].

North American Telles, formed by Metabolix Inc., among its PHB copolymer portfolio, has a food additive approved by the American Food and Drug Agency (FDA), the elastomer poly(3-hydroxyoctanate). The bacterium used for industrial production of this PHA is a genetically engineered *E. coli* K12, which can accumulate up to 90% (dcw) in just 24 h [98]. This high productivity in heterotrophic bacteria cheapens the industrial process, making this bioplastic accessible for large-scale production of disposables, such as combs, pens and other consumer goods.

PHB’s piezoelectric characteristic also makes it suitable for manufacturing electronics, such as computer equipment, microphones, detectors and sensors. The ability of some copolymers, such as PHBV, to barrier gases is useful in the application of food packaging, delaying the action of microorganisms in industrialized foods and drinks [6]. This is interesting for usage of PHB as a biomaterial in medical applications, as its piezoelectric characteristic promotes osteogenesis, assisting in bone regeneration [23,99,100]. The application of PHA in the medical field has been gaining attention, together with fine chemistry, because it deals with high value-added products and processes, and manages to better afford the high costs of PHA production at an industrial level. In addition the biocompatibility of these biopolymers makes them an interesting target for research and application in regenerative medicine, as well as in the sustained release of drugs and hormones [86,101], the PHAs most used for this purpose are: P(3HB), P(3HB-3HV), P(4HB), P(3HO) and P(3HB-3HHx), all of which have already been tested in vivo, showing biocompatibility [102]. 

Due to structural specificities, some polymers are more suitable for certain uses. As biomaterial, these PHAs have been reported to be useful in tissue engineering, bone orthoses and surgical sutures. PHB with its aforementioned piezoelectric characteristic is interesting for use in bone plates because it has a high biodegradation rate, in addition to being resistant to hydrolysis in sterile tissues [103]. Its high biodegradability also makes it ideal for the manufacture of surgical devices and medical material in general, that have short-use life. In tissue regeneration, microfilaments formed by copolymer P (3HB-co-3HV) maintained their masses and other characteristics for up to 12 months after being implanted in rats, being suitable for use as scaffolds [91]. Studies with PHB biofilms have shown a loss of up to 80% of the initial mass after one year of implantation [104]. The different results obtained regarding PHB degradation can be attributed to a series of chemical and physical factors. The enzymes present in the patient’s blood and tissues, for example, must be taken into account in the design of a biomaterial, as these can impair the rate of degradation [35,105].

The cardiovascular sector has benefited quite a lot from the application of PHA as a biomaterial: heart valves, pericardial adhesives, artery augers and implants in general are already available on the market, all from PHA, varying in their structures to better suit the final product [106]. In addition to contributions to the cardiovascular field, we mention P(4HB) for its importance as an exponent in medical biomaterials. Currently, this P(4HB) is the best option for use as a biomaterial compared to other thermoplastics available, being a strong biopolymer but with greater flexibility than synthetic absorbable polymers, such as polyglycolic acid (PGA) and polylactic acid (PLA), with a rate of bioabsorption of up to two years [107]. The sustained release of drugs is another possible application for PHA. In these systems, the drug or bioactive molecule is released gradually, without the need for new doses by the patient [86]. This approach is especially interesting for antibiotics, which maintain their therapeutic window, with a constant dosage of the drug, thus avoiding low dosage, where resistant strains of the pathogen could be selected.

Not only do PHA polymers have industrial applications, but also, the enzymes that produce them and their constituent monomers can be used in fine chemistry. PHA synthases are a good alternative for obtaining chiral compounds due to their enantioselectivity, they have specific action that can be used in the development of more specific chiral drugs, thus causing less adverse effects in patients, with the advantage of cleaner biosynthesis [108]. 3-hydroxybutyrate monomers can be used as precursors of new biopolymers, such as chiral polyesters—some with antibacterial, antiproliferative and hemolytic action—biocompatible dendrimers for carrying drugs and optically pure monomers, such as the ones that have already been used in the synthesis of sex hormones and fragrances [57,94,109].

## 3. Cyanobacteria

Known as blue algae, or cyanophytic algae, cyanobacteria are prokaryotic, Gram-negative, photosynthetic organisms of great biological importance, with fossils of cyanobacteria over 2.8 billion years [110,111,112]. Responsible for the oxygenation of the Earth’s atmosphere, these bacteria still play an important role in the carbon and nitrogen cycle on Earth [113,114], and they also originated chloroplasts, which were transferred horizontally to other strains and are now present in eukaryotes, namely higher plants [115]. The metabolism of cyanobacteria is noteworthy because they are the only microorganisms with photosynthesis similar to that of plants. They possess chlorophyll A, in addition to the pigment phycocyanin (a photosynthetic phycobilin), which gives the phylum its name, and its photosynthetic metabolism is also divided into two complementary photosystems, as it occurs in plants [116,117].

Cyanobacteria are organisms with high adaptive capacity which survive in environments with extreme temperatures, salinity, pH and levels of solar radiation [116,118,119]. Since photosynthesis is the main source of nutrition, these organisms can survive in almost any environment that has light, from dimly lit caves to open spaces with a high incidence of solar radiation [112]. They are also present in deserts, assisting in their fertility through nitrogen fixation [120]. The fixation of nitrogen in the environment by cyanobacteria is possible due to nitrogenase, an enzyme present in some species which makes them capable of assimilating unstable nitrogen gas (N_2_) as a direct source of nitrogen, in the form of more stable compounds such as nitrates, nitrites and ammonium salts. This makes them more independent in an environment with limited nitrogen [121]. A crucial factor for the functioning of the nitrogenase enzyme is its sensitivity to oxygen, which is a problem for cyanobacteria that produce oxygen via photosynthesis. As a solution, some cyanobacteria, such as those of the order Nostocales, developed specialized cells, the heterocysts, responsible for fixing nitrogen, spatially separating the two functions, while other orders simply operate photosynthesis and nitrogen fixation in different time periods [122]. 

Associating the photosynthetic process with nutrient metabolization, cyanobacteria are able to balance the electrons in their photosynthesis with their metabolism and thus neutralize the reduction caused by reactive oxygen species as a way of protecting against oxidative stress. Cyanobacteria UV-absorbing pigments also play a protective role [123,124]. The adaptive capacity of this phylum is explained in part by its production of secondary metabolites, which makes them competitive for survival in different environments. Such metabolites place cyanobacteria as highly promising microorganisms for biotechnological applications of commercial interest [125,126]. Compounds produced by cyanobacteria, which include fatty acids, amides, polyketides and lipopeptides, show several biological actions, including inhibition of glycosidases and of protein C kinases, tumor promoters, inhibitors of microtubulin aggregation [127] and immunosuppressive agents [128,129,130]. Some also have antibacterial, antialgal, antiplasmodial, antifungal and antiviral action, including anti-HIV, inhibiting human immunodeficiency virus synthesis [131].

In addition to application in segments with high added-value, such as the pharmaceutical market [132], the possibility of producing cyanobacterial biomass at a reduced cost allows its application in mass industries, which, as a rule, demand cheap production and on a large scale, such as the food and agriculture industry, with the use of cyanobacteria biomass and pigments as food and animal feed [133,134].

### 3.1. PHA Production in Cyanobacteria

The production of biopolymers is an example of the adaptation of cyanobacteria to environmental stimuli, especially nutritional deprivation, storing carbon in the form of glycogen and types of PHA produced by this phylum: PHB, PHV and their copolymers [12,135]. As PHB is the most prevalent polymer in cyanobacteria, we will focus on this short-chain bioplastic.

The accumulation of glycogen is a characteristic that is highly conserved, being found in a greater number of genera than the production of PHB or PHV [136]. Glycogen productivity is also higher than that of PHB, between 30% and 60% (dcw) [137], and glycogen is also produced quicker than PHB when the cell is deprived of nitrogen, being stored in several small granules in cells affected by stress [138]. Although the production of glycogen and PHB is activated by the same nutritional stress, the role of each polymer is different. In order to understand the role of each carbon storage, mutants of *Synechocystis* sp. PCC6803 with PHB and glycogen deficiency were compared [139]. In knockout mutants unable to produce glycogen, PHB accumulation increased between 8% and 13%, but most of the excess carbon was expelled from the cell in the form of pyruvate and α-ketoglutaric acid. These mutants were unable to turn on a dormant mode, that would save energy in the face of nutritional deprivation, and they also did not recover from nitrogen scarcity. PHB-deficient mutants, on the other hand, maintained the same rate of glycogen production as the wild-type, also maintaining recovery capacity once the nutrients were replenished. Only mutants that suffered knockout of genes related to both polymers showed deficiency in growth. 

This competition among the production of both polymers in cyanobacteria can be attributed to 3-phosphoglycerate (3PG) usage by PHB biosynthetic pathways as well as glycogen, with this last one making greater use of the 3PG pool obtained by cyanobacterial CO_2_ assimilation through Rubisco [7], hence for a robust PHB production, it is important to mitigate glycogen production [13]. Some authors compare PHB to triacylglycerol in green algae, since it also acts as an electron sink and consumes excess NADPH [138,139,140], while the storage of starch as an energy reserve in higher plants can be compared to the glycogen stock by cyanobacteria [139]. Although certainly important, the role of PHB in cyanobacteria has not been fully elucidated.

The first time PHB was reported in cyanobacteria was in 1966, in the single-celled *Chlorogloea fritschii*, which accumulated up to 10% (dcw) in PHB [141]. PHB and PHV have now been identified in different genera of cyanobacteria (Table 1).

The biosynthetic pathway for PHB production in cyanobacteria is shared with archaea and heterotrophic bacteria, in which three enzymatic reactions occur mediated by enzymes encoded by genes *phaA*, *phaB*, *phaC* and *phaE*. In cyanobacteria, PHA synthase coding is mediated by the last two genes, similar to that of anoxygenic purple sulfur bacteria [147,148,149]. The role of Rubisco in CO_2_ assimilation through the Calvin-Benson-Bassham cycle (CBB) [150], besides giving rise to the 3PG pool for glycogen and PHB production, is also a possible route for photosynthetic PHB production, with the 2-phosphoglycolate (2PG) resulting from CBB producing glycolate that in turn can be used as a starting blinding block for PHB synthesis. This was observed in a recombinant *Escherichia coli* JW2946 [151]. As for the production of PHV in cyanobacteria, it uses propionic acid as a starting point, and can occur in conjunction with PHB biosynthesis, resulting in the PHBV copolymer [12]. 

The production yield of these biopolymers in the literature in genus *Synechocystis* sp. ranges from 4% to 16% (dcw) under photo-autotrophy conditions [7,77,142,143]. Under heterotrophic conditions, higher yields are obtained, consistent with that obtained by other heterotrophic bacteria, although at lower levels, there is an accumulation of 28.8% (dcw) of PHB in the *Synechocystis* PCC6803 strain when cultivated with acetate supplementation and phosphorus deprivation [77]. Comparing wild and genetically modified organisms, production by *Synechocystis* sp. PCC6803 recombinant, grown only with acetate supplementation, showed a higher yield than that obtained previously, with an accumulation of 35% (dcw) of PHB [142]. The greatest yield in unicellular cyanobacteria was obtained with strain *Synechococcus* sp. MA19, reaching 55% (dcw), switching the phosphorus source for Ca_3_(PO_4_)_2_ [144].

An even greater production is observed in the filamentous cyanobacteria *Nostoc muscorum* Agardh, with an accumulation of 78% (dcw) in heterotrophy with nitrogen limitation, and supplementation of acetate, valerate and glucose [108]. Another N_2_-fixing cyanobacteria, *A. fertilissima*, under conditions of mixotrophic cultivation, with phosphorus and nitrogen deprivation and addition of citrate and acetate, showed an accumulation of 85% (dcw)—the highest obtained in cyanobacteria thus far [146].

### 3.2. Waste Utilization and Bioremediation

The use of agricultural residues as a carbon source or for supplementation of other nutrients has already been studied in wild and engineered organisms, such as heterotrophic bacteria and eukaryotic microalgae, in addition to cyanobacteria [48,152], being well assimilated by them in the production of metabolites for various industries, reducing input costs as well as agro-industrial waste. It is a logical path to use wastewater for the cultivation of cyanobacteria given their nutritional preferences, as the blooms of this phylum mostly occur in environments with an abundance of phosphorus and nitrogen, which are also widely found in aquaculture waste [153,154]. This strategy also makes it possible to save a key resource: water, which must have limited use in order to actually achieve a sustainable process [155].

Cyanobacteria and microalgae, in general, are ideal for removing nutrients and capturing CO_2_ from the most varied residues, reaching nutrient removal rates between 50% and 100% [15,36]. The main sources of nitrogen in sewage are metabolic interconversions of extra derived compounds, while about half of the phosphorus comes from detergents [154]. The ability of cyanobacteria to assimilate different forms of nitrogen and phosphorus, such as NH_4_+, NO_2_^−^, NO_3_- and PO_4_^3−^ [74], is well applied in wastewater treatment [156]. Sewage treatment is quite costly, in addition to being responsible for emissions to the environment [157,158], so bioremediation of these residues is an interesting alternative. Secondary sewage removal focuses on organic matter, and in order to reduce biological oxygen demand (BOD), this step benefits from heterotrophic bacteria that use said matter for growth and energy [154], but photosynthetic organisms also show potential for this role as they produce oxygen for other bacteria [159] and are also efficient in reducing oxygen demands in waste, as seen in the PHB-producing species *A. fertilissima* [78]. 

Secondary treated wastewater, although poorer in general as organic matter was mostly removed, is still loaded with micronutrients that can be further assimilated by cyanobacteria in a tertiary treatment [156,160]. *Phormidum* sp. is well applied in this sense because in addition to good nutrient removal rates, such as 48% of ammonium ions, 68% of total phosphate, 87% of nitrate and 100% removal of orthophosphate from swine wastewater [161], it has the capacity for self-aggregation, and this flocculation facilitates its later removal after bioremediation [162,163]. 

Pre-treated wastewater sewage was denitrified by a consortium of *Chlorella vulgaris*, *Botryococcus braunii* and *Spirulina platensis*, in a submerged membrane system in a photobioreactor, achieving good CO_2_ capture results and 92% removal of the initial 7.5 mg of total nitrogen N/L [164]. Wastes such as secondary effluents and digestate treatment with higher nitrogen and phosphorus content can, and should be used, since the higher concentration of these micronutrients results in dominance of cyanobacteria in the medium [163,165]. This is advantageous for the proposed application of bioremediation by cyanobacteria allied with industrial production of metabolites, providing an environment more suitable for cyanobacteria than other microalgae or heterotrophic bacteria [11,166]. Water reused directly from fish farming tanks was the nutritional source for the production of PHB by *Synechocystis* sp. 6803, reaching an accumulation of up to 20% (dcw) [77]. The aquaculture waste was characterized according to pH, available phosphorus and assimilable forms of nitrogen, separating the residues into two groups, both containing a lower concentration of phosphorus and nitrogen than conventional BG-11 medium.

Another work, besides using water from the aquaculture tank directly, minimizes processing steps, and proposes the diazotrophic cyanobacteria *A. fertilissima*, not only as a producer of PHB, but as a bioremediator of the water for aquaculture in a recirculatory system [78]. The greatest accumulation of PHB obtained was around 80% (dcw), achieved in the summer period [78]; in addition, this good result was accompanied by better bioremediation efficiency with all parameters within the accepted range for fish farming [167], with the lowest nitrate concentrations, total organic carbon and biological oxygen demand (BOD), as well as chemical oxygen demand (COD), occurring in this season. Ammonia, nitrite and orthophosphate were not even detected in this condition, being initially present in concentrations of about 2.3, 3.5 and 3 mg/liter respectively, illustrating the bioremediation capacity of *A. fertilissima*. The increase in dissolved oxygen promoted by this treatment makes fish less susceptible to ammonia toxicity [78], an additional benefit of this symbiosis.

More recently in *Nostoc muscorum* Agardh, the production of PHB was verified after supplementation with poultry waste [168]. Using 10 g/L of this agro-industrial waste, an increase of about 11% in PHB production was observed in relation to the control culture, with a total accumulation of 65% (dcw). This culture also received supplementation of 10% of CO_2_ in order to verify the capacity of this strain as a carbon dioxide mitigator, and therefore, adding yet another dimension in the search for sustainable production, reducing CO_2_ by means of solar energy using the electrons of photosynthesis [169,170]. CO_2_ fixation promoted a greater biomass production in *N. muscorum* and the same was verified in *S. platensis* [171], a species largely consumed as nutraceutical, with the goal of removing nutrients from pig farm waste, as a way to prevent eutrophication, reaching NH_4_-N removal of up to 95% and phosphorus removal rates up to 87%, respectively. Sludge from this kind of waste is rich in nutrients, with about 6 kg of nitrogen per ton of manure and 3 kg/ton of phosphorus [171,172]. Experimental culture media composed of this sludge is about 4.4 times cheaper than the usual Zarrouk culture medium [173].

## 4. Cyanobacteria Potential Application in Circular Economy

Despite the advantages PHA has over conventional plastics in terms of sustainability, for fossil plastic to be viably replaced, it is necessary to reduce the costs associated with the microbiological plastic production. Research and investments in the area have been making production cheaper. In 2002, the cost for manufacturing conventional petroleum-based plastic was €1.00/kg, a fraction of the PHA cost of €9.00/kg [24]. Two decades later, microbiological production of PHA can be obtained at €2.49/kg, which is still expensive, even compared to other sustainable polymers, such as PLA, costing €1.72/kg [9].

The main obstacles in the process concerns the carbon source used [50], the costs of maintaining the fermentation, the yield of the chosen inputs, the productivity and the downstream processing, including the extraction and purification of the polymer [10,11]. There are different strategies to face these obstacles; here, we will address only a few that are related to circular economy and industrial ecosystems, an approach that has already been applied with microalgae and heterotrophic bacteria [16,17,18,19,20]. The use of cyanobacteria is interesting because of the possibility of integrated production of different metabolites—with more than one type of compound as a salable product—and application of a “cradle-to-cradle” system [113], using by-products or production residues as a substrate for another product. Like the use of carbon monoxide (CO) in synthesis gas (syngas) for the production of PHB by the proteobacterium *Rhodospirillum rubrumde* [174], this author evens refers to this process as “grave-to-cradle”, turning a waste into a new product, bioplastic. Another example of waste being reapplied to the production process, now using microalgae, is the reuse of effluents from the refining of olive oil in the cultivation of microalgae for biodiesel and biopolymers [175]. This approach can benefit from the implementation and maintenance of an “inter-system ecology”, associating different industries [15,176].

From an environmental point of view, cyanobacteria are well-used as bioremediators, feeding on nutrients from domestic and agro-industrial waste, promoting nutrient removal and detoxification, removing heavy metals [78,164,177]. The assimilation of atmospheric carbon dioxide for conversion into biotechnological products [140,169] is another positive environmental impact, making the implementation of a circular bioeconomy more tangible. 

An alternative to make microbial PHB cheaper is to integrate the production of bioplastic with other desirable products, reusing by-products and residues of the microbiological production [14,178,179]. The production of acids for the cosmetic and pharmaceutical industry, such as eicosapentaenoic acid, by cyanobacteria of the genus *Nannochloropsis* sp., and γ-linoleic acid by cyanobacteria *Spirulina platensis,* is a viable alternative [180]. This species is also relevant for its expressive biomass production, with high protein content, suitable for application in nutraceuticals or animal feed [32,133]. The implementation of a biorefinery, integrating the PHB production of *Synechocystis salina*, with pigments of commercial interest, specifically phycocyanin and chlorophyll, commonly abundant in this phylum, and carotenoids, presented promising results [181]. The extraction of pigments without their degradation is not only possible, but essential, as the quality of the obtained polymer is directly affected by purification, which includes the removal of pigments, that can be used in production chains of higher value.

In addition to pigments, *S. salina* biomass has carbohydrates, lipids and proteins [181], which can be used for animal feed [134], provided that the necessary nutritional requirements and laws regarding the presence of contaminants such as heavy metals or mycotoxins are observed [182], and in this case, cyanotoxins [183], giving priority to non-toxin-producing cyanobacteria. The residual biomass of cyanobacteria would therefore be well-used in the nutrition of livestock and aquaculture, but it is possible to go further in the optimization of this production chain. Residues from these same livestock farming can be re-applied as supplementary nutrients to the growth of cyanobacteria in an integrated bio-factory [184]. The return of cyanobacterial by-products such as pigments and biomass to animal feed completes the proposed circular economy. Still, using *Spirulina* sp. as an example, its supplementation to animal feed has already been studied in shrimp, fish and chicken farming [134,185], valuing the production of this associated industry, improving the growth and coloring of tilapia [186] and egg yolks of chickens fed with *S. platensis* astaxanthin [187]. Animal health is also benefited by nutritional supplementation with cyanobacteria, with *Spirulina* sp. biomass improving the humoral and immunological response of chickens [129,130]. 

The dual advantage of production associated with bioremediation has already been described for cyanobacteria and microalgae in general, mainly aimed at the production of biodiesel [17,188,189,190]. The same concept can be applied to the production of biopolymers by cyanobacteria [15], naturally transformable organisms, which opens up possibilities for genetic engineering [143,191].

As a way to take advantage of Amazonian biodiversity in the search for microbial metabolites, in recent years, research with cyanobacteria from the Amazon has been developed with good results, and the sequencing of their genomes is an important tool in the search for compounds of biotechnological interest [192,193]. These organisms proved to be good producers of biodiesel, with yields higher than those in the literature and with parameters following international standards [194,195]. Biopolymer has also been detected in cyanobacteria in the region, with efforts being made to increase its production [196]. Subsequent work in this field would benefit from the approach proposed here of a circular economy, optimizing the resources employed, handling the waste and using its by-products and industrial “waste”, which is, as seen here, a potential feedstock for new biotechnological processes.

## 5. Conclusions

PHB-producing cyanobacteria, due to its lower productivity, in comparison to heterotrophic bacteria, are commercially viable only if combined with exploration of their various metabolites. Feed costs must be reduced, and using non-conventional feedstock, preferably agro-industrial waste, this opens up the possibility of industrial implementation within a circular economy, with the production of more than one product from this bio-factory, or with the use of by-products in other sectors, returning, for example, as animal nutrition to the industry itself, whose residues supplement cyanobacterial growth. Another great advantage of cyanobacteria in relation to other microorganisms producing PHB is its photosynthetic capacity, acting as a solar cell, reducing the CO_2_ of the environment, which is an assimilated carbon source and can be used as building blocks for the cyanobacterial production of bioplastic.

The critical reading of the studies already carried out in the area shows promising results for the manufacture and large-scale use of cyanobacterial biopolymers to be a reality in the near future, and more than that, demonstrates the need for cooperation between different knowledge and industries and through a circular economy to optimize the production process and reduce environmental impacts.

## Figures and Tables

**Table 1:** Examples of PHA-producing cyanobacteria, namely PHB and PHBV, with respective production in % (dcw) and nutritional conditions.

Cyanobacteria	Mode	Nutritional Deprivation	Nutritional Supplementation	PHA	Production % (dcw)	Reference
*Synechocystis* sp. PCC6803	Mixotrophic	P	Acetate	PHB	28.8	[77]
*Synechocystis* sp. PCC6803	Mixotrophic	N	Acetate	PHB	14.6	[77]
*Synechocystis* sp. PCC6803 (mutant)	Mixotrophic	-	Acetate	PHB	35	[142]
*Synechocystis* sp. PCC6803	Photoautotrophic	N, P	-	PHB	16.4	[143]
*Synechococcus* sp. MA19	Photoautotrophic	P	-	PHB	55	[144]
*Nostoc muscorum* Agardh	Mixotrophic	N	Glucose, acetate, valerate	PHBV	78	[145]
*Chlorogloea fritschii*	Mixotrophic	-	Acetate	PHB	10	[141]
*Spirulina subsalsa*	Photoautotrophic	N	-	PHB	14.7	[32]
*Aulosira fertilissima*	Mixotrophic	N, P	Acetate, citrate	PHB	85	[146]

P = phosphorus; N = nitrogen.
